# Perspectives on the Impacts of a Food Support Program on the Well‐Being of People Living With HIV (PLHIV) and on Antiretroviral Therapy (ART) in Khomas Region, Namibia

**DOI:** 10.1155/arat/6201140

**Published:** 2026-07-01

**Authors:** Linda Ndeshipandula Lukolo, Martin Shapi, Edith Hamukwaya

**Affiliations:** ^1^ Department of Health Sciences, School of Medicine, Faculty of Health and Veterinary Sciences, University of Namibia, Windhoek, Namibia, unam.edu.na; ^2^ Multidisciplinary Research Services, University of Namibia, Windhoek, Namibia, unam.na

**Keywords:** food support program, human immunodeficiency virus (HIV), Namibia, nutritional support, qualitative study

## Abstract

**Background:**

People living with HIV (PLHIV) often face nutritional deficiencies resulting from reduced food intake, malabsorption, and increased metabolic demands. Adequate nutrition is essential for optimizing antiretroviral (ARV) drug absorption, reducing treatment side effects, managing HIV‐related malnutrition, and supporting immune recovery. In 2012, the Khomas Regional Council introduced a food support program for PLHIV on antiretroviral therapy (ART); however, its impact has not been systematically evaluated. This study aims to explore key informants’ perspectives on the program’s impact on the well‐being of PLHIV receiving ART in the Khomas Region, Windhoek, Namibia.

**Methods:**

A qualitative phenomenological design was employed. Sixteen purposively selected key informants from eight constituencies in the Khomas Region participated in in‐depth, semistructured interviews. Data collection occurred in two phases: June–August 2024 (*n* = 8) and October 2025 (*n* = 8). Interviews followed a guiding framework with probing questions, and data saturation determined sample adequacy. Ethical procedures, including informed consent, confidentiality, and the protection of participants’ rights, were rigorously upheld.

**Results:**

Three overarching themes emerged: (1) Positive impacts of the food support program, including noticeable weight gain among beneficiaries, improved ART adherence, reduced ART dropout rates, and increased confidence among PLHIV; (2) challenges affecting the program implementation, which included insufficient food supplies, limited funding resources, migration of beneficiaries, lack of transport for field workers, and persistent self‐stigma among PLHIV; and (3) Strategies for strengthening the program, such as increasing the quantity of food provided, ensuring consistent and frequent food distribution, promoting income‐generating activities, updating the beneficiary database, and intensifying efforts to address HIV‐related stigma.

**Conclusions:**

Despite challenges, the food support program positively influenced the health and well‐being of PLHIV. Strengthened collaboration between the Khomas Regional Council and ART clinics as well as awareness campaigns are recommended to broaden the program’s reach.

## 1. Introduction

Human Immunodeficiency Virus (HIV) continues to pose a significant public health challenge globally, with sub‐Saharan Africa bearing the highest burden. The widespread availability of antiretroviral therapy (ART) has transformed HIV from a life‐threatening condition to a manageable chronic disease, improving the quality of life and life expectancy of people living with HIV (PLHIV) (World Health Organization [1]. However, optimal health outcomes depend on more than just ART access; social factors such as food security play a crucial role in treatment adherence and overall well‐being [2]. Namibia is among the countries most affected by HIV, despite significant progress in prevention, treatment, and care. Recent national estimates indicate that HIV prevalence among adults aged 15–49 years is approximately 9%–9.7%, reflecting a gradual decline over the past decade Centers for Disease Control and Prevention [3, 4]. As of 2023, an estimated 197,651 people were receiving ART in Namibia, demonstrating substantial expansion in treatment coverage and progress toward achieving global HIV targets [3]. Additionally, Namibia has made notable strides toward the UNAIDS 95–95–95 targets, with high levels of diagnosis, treatment uptake, and viral suppression reported nationally [5]. Despite these achievements, HIV remains highly prevalent, particularly among women and vulnerable populations. According to recent national reports, approximately 228,538 people are living with HIV in Namibia, with women and girls accounting for about 65% of all cases, highlighting persistent gender disparities in HIV infection rates ([5]; Namibia Ministry of Health and Social Services [6]. While treatment access has improved significantly, challenges such as poverty, inequality, and food insecurity continue to undermine optimal health outcomes among PLHIV.

The Khomas Region, which includes Namibia’s capital city, Windhoek, is a critical hub for HIV treatment and care services. With a population exceeding 431,000, the region hosts a large proportion of PLHIV and provides extensive ART services through hospitals, health centers, and primary healthcare clinics [6]. Evidence from facility‐based data indicates that at least 20,173 patients were actively receiving ART in Windhoek alone in 2022, illustrating the substantial treatment burden within the region [7]. Earlier reports also suggest that over 24,000 individuals in the Khomas Region were on ART, underscoring the sustained demand for HIV treatment services over time [8]. Although ART coverage is high, retention in care and long‐term adherence remain ongoing concerns, particularly among adolescents and socioeconomically disadvantaged populations [9].

Food insecurity and undernutrition are common among PLHIV in Namibia and can undermine treatment effectiveness because inadequate nutrition increases drug side effects, reduces tolerance of medication, and is associated with poorer adherence and clinical outcomes [10]. A landmark local study conducted in Windhoek found severe household food insecurity in a large proportion of adults living with HIV attending public ART clinics and reported a clear association between food insecurity and poorer ART adherence (measured by medication possession ratio [MPR] [cite]). This finding underscores the direct link between household food access and treatment continuity for PLHIV in the Khomas Region [11]. Food insecurity refers to the lack of regular access to sufficient, safe, and nutritious food needed for an active and healthy life at individual or household level due to a lack of money or other resources [12]. Studies conducted in Namibia found that 67% of PLHIV attending public ART clinics experienced severe household food insecurity, which was associated with poor ART adherence, as measured by the MPR [13, 14], Similarly, a case–control study performed in Northern Ethiopia found that malnutrition (BMI < 18.5), poor dietary patterns, and other nutritional factors were significantly associated with nonadherence to ART [15]. The mechanisms through which food insecurity affects ART adherence are multifaceted. Qualitative studies have highlighted that PLHIV often skip or delay taking ART due to the exacerbation of side effects when medications are taken without food, the belief that ART should not be taken on an empty stomach, and the prioritization of limited resources for food over transportation to clinics [15, 16]. These challenges underscore the need for comprehensive interventions that address both medical and socioeconomic determinants of health [11].

Food support programs (FSPs) have been implemented to mitigate the impact of food insecurity on ART adherence. For instance, in Lusaka, Zambia, studies demonstrated that providing food supplementation to PLHIV on ART significantly improved adherence rates, as well as their nutritional status, with 70% of patients in the food support group achieving an MPR of 95% or greater, compared to 48% among controls [17]. Similar evidence from sub‐Saharan Africa shows that food assistance is associated with improved ART adherence and retention in care, as well as enhanced clinical outcomes among PLHIV [18, 19]. These findings highlight the critical role of integrating food support into HIV treatment programs to address structural barriers to adherence.

These findings suggest that integrating nutritional support into HIV care can enhance treatment outcomes. Despite the recognized importance of FSPs, there is a paucity of research exploring key informants’ perspectives on the impact of such interventions on the well‐being of PLHIV, particularly in the Namibian context. Understanding the experiences and perceptions of stakeholders, including program beneficiaries, healthcare providers, and policymakers, is crucial for informing the design and implementation of effective food support initiatives. This study aims to assess key informants’ perspectives on the impact of an FSP on the well‐being of PLHIV receiving ART in the Khomas Region of Namibia. By employing a qualitative phenomenological approach, the research seeks to elucidate the perceived benefits, challenges, and recommendations for enhancing the effectiveness of FSPs in improving health outcomes for PLHIV.

## 2. Methods

### 2.1. Study Design

This study employed a qualitative research method to gain insights into the perceived impact of the FSP on the lives of PLHIV who are on ART in the Khomas Region of Namibia. A descriptive phenomenological design was used to explore lived experiences and subjective perceptions of key stakeholders involved in the implementation and coordination of the program. Qualitative methods are particularly suitable for exploring complex social and health‐related issues, allowing participants to freely express their experiences and providing nuanced insights into human behavior and perceptions [20, 21].

### 2.2. Study Setting and Population

**FIGURE 1:**
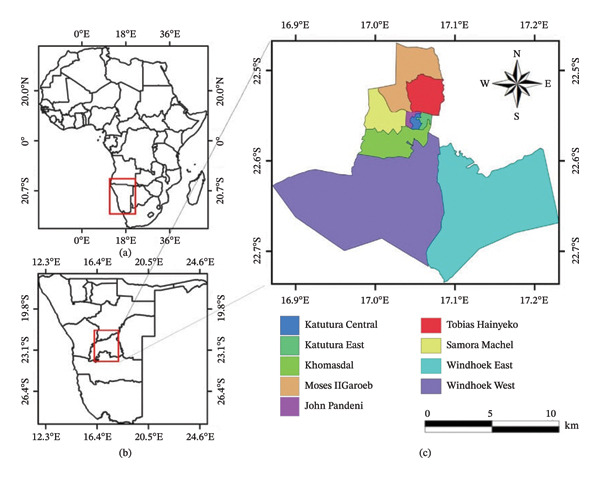
A map of Namibia showing Khomas Region constituencies: (a) location of Namibia in Africa, (b) location of Windhoek in Namibia, and (c) Khomas Region and constituencies.

The study was conducted in Windhoek, the capital city of Namibia, located in the Khomas Region. Khomas Region is centrally situated in Namibia and includes the capital city, Windhoek, making it the country’s most populous region. It consists of 10 constituencies, of which eight were purposively selected for this study, as these are the areas where the FSP for PLHIV on ART has been introduced. The selected constituencies are John Pandeni, Katutura Central, Katutura East, Samora Machel, Moses ǁGaroëb, Tobias Hainyeko, Khomasdal, and Windhoek West; see Figure 1. The Khomas Region has a mix of formal and informal settlements; recent census and population estimates show the region’s population in the hundreds of thousands, with rapid urban growth concentrated in Windhoek. Although Khomas is urban, it contains large informal settlements such as Katutura, Samora Machel, Havana, and Okuryangava, where poverty, unemployment, and food insecurity are most widespread. Many households depend on irregular income from informal work. For example, selling street food (vetkoek or Kapana) at local markets or mobile street vendors, casual construction work, and their daily earnings vary day by day. On busy days, they may earn better; however, on slow days, they may earn little or nothing, making consistent access to nutritious food difficult. People on ART in these communities frequently report challenges meeting the nutritional requirements needed to support treatment (PHIA/[22]).

The Khomas Region was selected for the FSP because it hosts the highest concentration of people on ART in Namibia, with a regional population of 494,604, an HIV prevalence of 8.3%, and more than 24,000 individuals currently receiving ARVs [23]. The selected constituencies in Windhoek’s informal settlements face persistent socioeconomic challenges, including high levels of poverty and food insecurity, which negatively affect treatment adherence among PLHIV. The FSP, implemented by the Khomas Regional Council (KRC), provides targeted nutritional assistance to vulnerable PLHIV enrolled on ART to strengthen adherence, improve clinical outcomes, and support overall well‐being. Vulnerable PLHIV refers to individuals on ART who, due to food insecurity, poverty, clinical instability, or psychosocial challenges, are at a heightened risk of poor adherence or adverse health outcomes and thus qualify for nutritional support [24]. Given its demographic diversity, high mobility, and the socioeconomic vulnerabilities affecting PLHIV, Khomas offers an ideal setting for examining the influence of food support interventions on treatment adherence and holistic well‐being. Figure 1 depicts the map of Namibian regions, the Khomas Region, and the constituencies of Windhoek.

### 2.3. Participants and Sampling

Key informants were purposively selected based on their professional roles within the FSP and their direct interaction with PLHIV enrolled in ART. These informants included community health workers, program implementers, and constituency‐level coordinators across the Khomas Region. Their responsibilities such as food distribution, maintaining beneficiaries’ weight‐monitoring records, and conducting home follow‐up visits positioned them as rich sources of experiential insights relevant to the study. A total of 16 participants, age range between 38 and 55 years old, were recruited, representing eight constituencies of the Khomas Region, as shown in Figure 1. Purposive sampling, guided by maximum variation principles, was employed to ensure inclusion of diverse viewpoints across constituencies, job roles, and program responsibilities. Participants were identified in collaboration with staff from the KRC, who facilitated access to individuals actively involved in the day‐to‐day implementation of the program. To qualify to be a key informant, the person should be in the program for 2 years or more.

### 2.4. Data Collection

Data were collected in two phases: June–August 2024 and October 2025, respectively, using in‐depth, semistructured phenomenological interviews designed to capture the lived experiences and perspectives of key informants involved in the FSP. This approach allowed participants to describe, in their own words, how the program is implemented, its perceived impact on beneficiaries’ health and treatment adherence, and the operational challenges encountered across constituencies. All interviews were conducted in a private and confidential setting to ensure comfort and openness. With participants’ informed consent, interviews were audio‐recorded to preserve the accuracy and richness of the narratives. An interview guide with open‐ended questions was used to steer the discussion, while follow‐up probing questions enabled deeper exploration of emerging insights and experiences. Interviews were conducted face‐to‐face in the language preferred by each participant, English, Oshiwambo, or Afrikaans, and each session lasted approximately 45–60 min. In addition to the audio recordings, detailed field notes were kept to document contextual factors, nonverbal cues, and the researcher’s reflexive observations. These notes contributed to a more holistic and nuanced phenomenological interpretation of the data. Recruitment continued until data saturation was achieved, that is, when no new information, themes, or perspectives emerged from subsequent interviews [25, 26]. This approach strengthened the credibility and depth of the study findings.

### 2.5. Data Analysis

All interview recordings were transcribed verbatim, and translations into English were completed where required to ensure accuracy and preservation of meaning. Data were analyzed using Braun and Clarke’s reflexive thematic analysis framework (cite), which provides a systematic yet flexible approach suitable for phenomenological inquiry. The six‐phase process involved (1) immersing in the data to achieve familiarization, (2) generating initial codes, (3) searching for potential themes, (4) reviewing and refining themes, (5) defining and naming themes, and (6) producing a coherent analytical narrative. Manual coding was conducted independently by two researchers to promote rigor, reduce potential bias, and enhance coding consistency. The researchers then collaboratively compared and reconciled codes, merged related categories, and finalized themes and subthemes through iterative discussion. This collaborative process strengthened the credibility, dependability, and trustworthiness of the analytical outcomes. A reflexive approach was maintained throughout to ensure that interpretations were grounded in participants’ lived experiences and contextual realities (Manuel, Carlos & Beatriz, 2025).

### 2.6. Data Storage and Security

Audio recordings, transcripts, and field notes were stored in encrypted, password‐protected digital folders on secure institutional servers. Only the principal investigator and authorized research team members had access to the data to uphold confidentiality, given the sensitivity of HIV‐related topics. All raw files were backed up on a secure, encrypted cloud system to prevent accidental loss.

### 2.7. Trustworthiness

To ensure the quality and rigor of the qualitative findings, the study adhered to the established criteria of credibility, transferability, dependability, and confirmability.

Credibility was strengthened through prolonged engagement with participants during interviews, the use of probing questions to elicit depth, and the triangulation of information from participants working across different constituencies. Independent coding by two researchers, followed by collaborative discussions to reconcile differences, further enhanced the accuracy and authenticity of the interpretations. Transferability was supported by providing rich, detailed descriptions of the study setting, participant characteristics, and contextual factors surrounding the FSP. These detailed accounts enable readers to assess the applicability of the findings to similar contexts. Dependability was ensured through maintaining a clear audit trail documenting methodological decisions, coding procedures, and analytical steps. Consistent use of Braun and Clarke’s thematic analysis framework contributed to a stable and transparent analytical process. Confirmability was promoted by practicing reflexivity throughout the study. The researchers maintained field notes and reflexive memos to identify personal biases and ensure that interpretations remained grounded in participants’ perspectives rather than the researcher’s assumptions [27].

### 2.8. Ethical Considerations

This study followed established ethical principles for research involving human participants. Ethical approval was obtained from the University of Namibia Human Research Ethics Committee (HREC) [OSHAC/595/2022], the review board, and the KRC prior to data collection. Permission to access program implementers and constituency‐level staff was secured through the appropriate administrative channels. Informed consent was obtained from all participants before their involvement in the study. Participants were informed of the study’s purpose, procedures, potential risks, and their right to withdraw at any time without negative consequences. Confidentiality and anonymity were rigorously maintained by using unique identifiers instead of personal names and by securely storing interview recordings, transcripts, and related documents. Interviews were conducted in private settings to ensure comfort and confidentiality. Audio recordings and transcripts were stored on password‐protected devices accessible only to the research team. All data were preserved and managed in accordance with institutional data protection guidelines. The study adhered to the principles of respect, beneficence, and justice, ensuring that participants’ rights, dignity, and well‐being were prioritized throughout the research process [27].

## 3. Results and Discussion

This section presents the findings derived from sixteen in‐depth interviews conducted with key informants involved in the implementation of the FSP. Through systematic thematic analysis, three overarching themes and their associated subthemes were identified. These themes reflect participants’ experiences and perspectives regarding: (1) the positive impacts of the FSP, (2) challenges affecting program implementation, and (3) proposed strategies and pathways for strengthening the program moving forward.

A detailed summary of the themes and subthemes emerging from the data is provided in Table 1, which serves as an overview of the key insights explored in the subsequent sections.

**TABLE 1:** Themes and subthemes.

Themes	Subthemes
The positive impacts of the food support program.	➢ Observable weight gain among beneficiaries
	➢ Reduced rate of treatment defaulting and improved ART adherence
	➢ Enhanced confidence and overall psychosocial well‐being as well as program sustainability

Challenges affecting program implementation	➢ Insufficient food support
	➢ Limited quantity of food items
	➢ Inadequate nutritional quality
	➢ Limited financial resources and budget constraints
	➢ Frequent migration of beneficiaries
	➢ Lack of transport for field workers conducting follow‐up
	➢ Presence of self‐stigma among PLHIV

Proposed strategies and pathways for strengthening the program moving forward.	➢ Increase both the quantity and nutrition quality of food parcels
	➢ Promote awareness and engagement in income‐generating activities (e.g., small‐scale or backyard gardening)
	➢ Regularly update and maintain an accurate beneficiary database

## 4. Themes and Subthemes

### 4.1. Theme 1: Positive Impacts of the FSP

The first theme highlights the beneficial effects of the FSP on the health and overall well‐being of PLHIV enrolled in ART within the Khomas Region. Participants consistently reported visible improvements among beneficiaries, particularly in terms of nutritional status, treatment adherence, and renewed physical functioning. These positive outcomes underscore the value of food support as a complementary intervention in HIV care. Table 1 summarizes the associated subthemes.

#### 4.1.1. Subtheme 1: Observable Weight Gain and Improved Physical Health

Across the interviews, participants frequently noted that beneficiaries experienced noticeable weight gain after receiving regular food parcels. This improvement was regarded as a strong indicator of better nutritional intake and enhanced immune function. Many key informants described how beneficiaries who were previously weak, fatigued, or bedridden regained strength and became more active in their daily lives.

Participants explained:

“The program is really helping because some of our beneficiaries gained weight after receiving the food parcel. You can see that they have energy now compared to the first day of receiving food.” (Stakeholder—Katutura Central)

Participants shared similar observations:

“Some beneficiaries were bedridden, but now they are doing good. They look healthy; some have even gone back to their work or doing their daily routine activities.” (Stakeholder—John Pandeni)

These accounts emphasize the critical link between nutrition and improved health outcomes among PLHIV. Adequate food intake plays a vital role in strengthening the immune system, supporting ART effectiveness, and enhancing physical resilience. This is consistent with the study by Rome [28], who asserted that in PLHIV, good nutrition supports overall health, helps maintain immune function, and improves the body’s ability to tolerate medication. Improved nutrition therefore not only contributes to better health status but also enhances beneficiaries’ capacity to participate more fully in social and economic activities [17, 29].

#### 4.1.2. Subtheme 2: Reduced Treatment Defaulting and Improved ART Adherence

Another key positive outcome identified in the study was the improvement in ART adherence among beneficiaries receiving food support. Participants noted that consistent access to food enabled beneficiaries to take their medication regularly, as many reported struggling with adherence when taking ART on an empty stomach. Although no formal clinical adherence assessment was conducted, participants described clear improvements based on their observations and routine monitoring of clients’ health passports during home visits.

Participants explained:

“The rate of defaulting dropped. No study was done on this, but from my observation and from asking them. I always ask them to come with their health passport so I can check if they are really attending their follow‐up at the clinics or hospital.” (Stakeholders—Samora Machel, Katutura East).

Some participants added:

“The food helps them to take their medications every day.” (Stakeholders—Tobias Hainyeko, Windhoek Central)

These observations align with evidence showing that food insecurity is strongly associated with poor ART adherence and that nutrition support can significantly enhance consistent medication‐taking behavior. Studies have demonstrated that food assistance improves treatment adherence, boosts energy levels, and strengthens beneficiaries’ ability to maintain daily routines [30, 31].

#### 4.1.3. Subtheme 3: Improved ART Adherence, Psychosocial Well‐Being, and Program Sustainability

In addition to physical and clinical benefits, participants highlighted important psychosocial improvements among beneficiaries. The FSP contributed to enhanced confidence, improved self‐worth, and greater social interaction. Stakeholders reported that receiving food strengthened beneficiaries’ sense of dignity and reduced feelings of dependence, as many felt empowered to provide for their households. The FSP was reported to improve ART adherence among beneficiaries. Participants observed that access to food enabled PLHIV to take their medication consistently, particularly as taking ART on an empty stomach was previously a barrier. Participants noted:

“The rate of defaulting dropped. I always ask them to bring their health passport to check if they are attending their follow‐ups at the clinic.” (Stakeholders—Katutura Central, John Panfeni, Moses Garoeb)

Some participants added:

“The food helps them to take their medications every day.” (Stakeholders—Katutura Rural, Samora Machel)

These findings are supported by evidence indicating that nutrition supplementation and food assistance significantly enhance adherence, nutritional status, and overall quality of life for PLHIV [31, 32]. Participants also highlighted improvements in psychosocial well‐being and confidence. Receiving food fostered a sense of pride and ownership, with beneficiaries feeling empowered to provide for their households:

“They feel confident by getting food because they feel now that they are breadwinners for the house. ‘They can put food on the table and feel proud.’” Stakeholders—Samora Machel, Katutura Central)

“Beneficiaries proudly call the food program ‘food for us.’” (Stakeholders—John Pandeni, Tobis Hainyeko, Katutura East)

Regarding program sustainability, key informants emphasized the role of multistakeholder collaboration, including the KRC, Regional AIDS Coordinators, field workers, TCE, and the Ministry of Health and Social Services, to ensure continuous identification and recruitment of beneficiaries. This strong institutional and community support has contributed to the program’s ongoing operation since 2012. These findings are consistent with the broader literature indicating that food support not only improves nutritional and health outcomes but also enhances psychosocial well‐being, reduces stigma, and fosters a stronger sense of community among PLHIV [33, 34]. Although primarily intended for PLHIV, participants reported extending support to nutritionally vulnerable TB patients, recognizing the importance of adequate food for medication tolerance and recovery:

“When a person with TB comes to the office hungry, we cannot turn them away unless there is no food in stock.” (Stakeholders—Windhoek West, Moses Garoeb)

Balanced nutrition is critical for TB treatment and recovery, as proper dietary intake supports the body’s ability to fight infection and tolerate medication [31, 35].

### 4.2. Theme 2: Challenges Affecting the FSP

Although the FSP plays a vital role in improving the well‐being of PLHIV on ART, several challenges threaten its effectiveness and sustainability. These challenges are outlined below.

#### 4.2.1. Subtheme 1: Limited Quantity and Nutritional Inadequacy of Food Items

Key informants consistently emphasized that the food supplied is often insufficient—both in quantity and nutritional quality—and distribution is irregular. These constraints negatively affect beneficiaries’ ability to maintain adequate dietary intake while adhering to ART.

Participants explained:

**“**The program is helpful, however the food we receive from the regional office is not enough. Sometimes beneficiaries come here and there is no food. You feel so bad seeing someone hungry going back with nothing—we even end up giving ten dollars from our pocket for bread.” (Stakeholders—Katutura East, Katutura Central, John Pandeni, Samora Michel, John Pandeni)

Some participants added:

**“**It is a concern for someone who wants to take medication but there is no food. Sufficient food is crucial for our beneficiaries’ health.” (Stakeholders—Windhoek East, Tobias Hainyeko)

These sentiments reflect broader evidence that adequate nutrition is essential for immune function and ART effectiveness. Studies report that food insecurity is strongly linked to poor ART adherence, weakened immunity, and higher morbidity among PLHIV [36, 37]. Similarly, de Pee and Semba [38] argue that food insecurity can reduce treatment initiation and adherence by limiting energy intake and worsening medication side effects.

#### 4.2.2. Subtheme 2: Limited Financial Resources and Funding Constraints

A major barrier to program sustainability stems from inadequate financial resources. The KRC remains the sole funder, despite high demand and rising food insecurity levels.

As participants indicated:

“Attending to people in need with few resources is disappointing. There is no sufficient money to buy food.” (Stakeholders—Windhoek Central, Windhoek East, John Pandeni, Samora Machel, Moses Garoeb)

“Khomas Regional Office is the only one supporting the program financially despite the high demand.” (Stakeholders—Windhoek Central, Katutura Central Katutura East, Tobias Hainyeko)

Due to funding gaps, the program relies heavily on stakeholders such as MoHSS, AIDS coordinators, and field workers to identify and support beneficiaries.

Participants recommended diversifying funding sources:

“The regional council should approach NGOs, business communities, even the Ministry of Fisheries for donations—fish is nutritious.” (Stakeholders—Samora Machel, John Pandeni, Katutura Central)

This aligns with recent recommendations emphasizing the need for multisectoral funding and partnerships to strengthen HIV–nutrition support programs [39, 40].

#### 4.2.3. Subtheme 3: Frequent Migration of Beneficiaries

Constant movement of beneficiaries poses a significant tracking and follow‐up challenge. Migration interrupts food distribution schedules and complicates case management.

Participants shared:

**“**The problem we experience is migration. Beneficiaries move from one place to another without notifying us—you go for a visit only to find they no longer stay there.” (Stakeholders—John Pandeni, Samora Machel, Windhoek West, Moses Garoeb)

Participants added:

“This issue is out of our control; we cannot restrict their movements.” (Stakeholders—Samora Machel, Katutura Central, Windhoek East)

Migration among PLHIV is widely documented as a barrier to continuity of care, resulting in missed appointments, treatment interruptions, and poor monitoring outcomes [1, 41, 42].

#### 4.2.4. Subtheme 4: Lack of Transport for Fieldworkers Conducting Follow‐Up

Fieldworkers responsible for follow‐up face logistical difficulties accessing beneficiaries in remote informal settlements.

This challenge was expressed as:

“Yes, one of the challenges is long distance to reach beneficiaries and there is no transport for fieldworkers. They end up not reaching far areas.” (Stakeholders—Windhoek West, Katutura Central)

Inadequate transport reduces monitoring efficiency and delays support for vulnerable households—similar to findings from regional studies showing that logistical constraints impede HIV–nutrition outreach programs [40, 43].

#### 4.2.5. Subtheme 5: Presence of Self‐Stigma Among PLHIV

Self‐stigma remains a major obstacle in the identification and registration of eligible beneficiaries. Some PLHIV avoid seeking assistance due to fear of disclosure, judgment, or social isolation.

Participants reported:

“Many people living with HIV are still hiding and suffering in isolation. They are not coming out.” (Stakeholder—John Pandeni)

“There are people who need to be registered, but they are not coming out—maybe they fear being known.” (Stakeholders—Moses Garoeb, John Pandeni)

Research shows that self‐stigma reduces help‐seeking behavior, adherence, and participation in support programs [45]. Misconceptions about HIV transmission and outdated beliefs continue to fuel stigma in communities [3, 46]. Encouraging open dialog, community education, and psychosocial support are essential steps toward reducing self‐stigma.

### 4.3. Theme 3: Proposed Strategies and Pathways for Strengthening the FSP

While the FSP plays a vital role in the well‐being of PLHIV on ART, key informants identified several strategies to improve its effectiveness, sustainability, and long‐term impact. These proposed pathways aim to strengthen program delivery, enhance nutritional outcomes, and reduce dependency.

#### 4.3.1. Subtheme 1: Increasing Both the Quantity and Quality of Food Parcels

Key informants stressed the need to expand both the volume and nutritional adequacy of the food parcels. The current rations were described as insufficient to meet the nutritional demands of PLHIV, who require higher caloric and micronutrient intake due to increased metabolic activity and malabsorption caused by HIV.

Participants emphasized:

**“**The amount of food is too little for three to four months. The government should give enough food to people living with HIV, especially those who cannot feed themselves.” (Samora Machel, John Pandeni, Katutura East and Central, Windhoek East and West, Moses Garoeb, Tobias Hainyeko)

Many of the participants highlighted the need for consistency:

“The frequency of receiving food should be increased—at least once every month—and distribution must be consistent.” (Stakeholders—all)

These concerns align with global evidence showing that PLHIV require increased nutritional support due to the effects of HIV on appetite, nutrient absorption, and energy expenditure [37, 42]. Research confirms that adequate nutrient intake improves immune function, reduces HIV‐related complications, and enhances ART adherence ([48, 49], 2018 [50]).

#### 4.3.2. Subtheme 2: Promoting Awareness and Engagement in Income‐Generating Activities (e.g., Backyard or Small‐Scale Gardening)

Participants recommended empowering beneficiaries through income‐generating activities, especially small‐scale or backyard gardening. This approach would improve household food security, encourage self‐reliance, and reduce pressure on the program.

Participant expressed:

**“**Beneficiaries should be encouraged to start small projects like gardening. The government alone cannot meet all their needs—they need awareness on income‐generating activities.” (Stakeholders—Katutura East and Central, Windhoek East)

Another added:

“Mmmmh… I think it will help if beneficiaries do small gardening at the backyard or tyre gardening. They can grow spinach, tomatoes, onions. This will prevent dependency.” (Stakeholders—Samora Machel, Katutura Central, Windhoek East)

Some participants highlighted the need for support:

“These people just need training and maybe seeds to help them grow their vegetables and fruits.”(Stakeholders—Samora Machel, Katutura Central, John Pandeni, Windhoek East)

Recent literature supports home gardening and small‐scale agriculture as effective strategies for improving dietary diversity, reducing food insecurity, and enhancing resilience among PLHIV and low‐income households [39, 51]. Locally grown produce is often fresher, more nutritious, and free from long supply‐chain delays or excessive chemicals. Furthermore, community‐based livelihood programs have been linked to improved ART adherence, psychosocial well‐being, and long‐term sustainability of HIV‐support interventions [40, 52].

#### 4.3.3. Subtheme 3: Regular Updating and Maintenance of an Accurate Beneficiary Database

The study revealed inconsistencies and outdated information in the beneficiary database, including missing beneficiaries, unrecorded relocations, and incomplete constituency lists. Such gaps hinder effective planning, monitoring, and targeted distribution.

Some participants explained:

“The database is not up to date some beneficiaries are not on the constituency list, and some have already left the area.” (Stakeholders—Samora Machel, Katutura Central, Windhoek East)

Some participants added:

“The list needs to be updated regularly. It will help the program run smoothly and ensure food reaches the right people.” (Stakeholders—Tobias Hainyeko, Katutura Central, Windhoek East)

Accurate data are essential for resource allocation, monitoring treatment adherence, and ensuring that the most vulnerable are adequately supported. Recent studies emphasize that digital tracking systems and continuous household monitoring improve the efficiency and accountability of food and HIV‐support programs [53, 54].

Implementing routine updates, strengthening coordination among fieldworkers, and adopting digital tools can greatly enhance program targeting and reduce administrative gaps.

#### 4.3.4. Study Limitations and Strengths

This study was limited by its relatively small sample size, which may restrict the generalizability of the findings beyond the Khomas Region. The inclusion of beneficiaries could have given more information. Self‐reported information from participants may also introduce recall or social desirability bias. Additionally, the study focused only on beneficiaries currently enrolled in the FSP, which may exclude the perspectives of eligible individuals who are not accessing the service. Time and resource constraints further limited the depth of follow‐up with participants. Despite these limitations, the study offers important strengths. It provides rich, context‐specific insights derived directly from key informants, working with PLHIV, by distributing food, conducting follow‐up home visits, and providing and receiving nutritional support. The use of qualitative methods allowed for an in‐depth understanding of lived experiences, program benefits, and challenges within a real‐world setting. The study also contributes locally relevant evidence that can inform future program improvements and guide policy decisions in Namibia’s HIV and nutrition support landscape.

## 5. Conclusion

The study demonstrates that the FSP plays a significant role in improving the health, treatment adherence, and overall well‐being of PLHIV in the Khomas Region. Access to reliable and nutritious food supports immune function, enhances ART effectiveness, and strengthens beneficiaries’ confidence and ability to manage their daily lives. However, challenges such as limited food quantity and quality, funding constraints, beneficiary migration, self‐stigma, and inadequate transport for fieldworkers hinder the program’s full impact. Strengthening partnerships, improving service delivery systems, and expanding community awareness are essential for ensuring that the program remains sustainable and responsive to the needs of PLHIV. By addressing these challenges and enhancing program accessibility including through stigma‐free registration and home‐based support KRC and its partners can ensure that all eligible individuals receive the nutritional assistance necessary to maintain their health and quality of life. The study recommends the following.

### 5.1. Strengthen Multisector Collaboration

The findings highlight that the KRC is currently the sole financial supporter of the FSP. It is recommended that the Council proactively engage NGOs, private companies, humanitarian agencies, and international donors to expand funding and resource support as beneficiary needs continue to grow.

### 5.2. Enhance Community Awareness and Outreach

Awareness of the FSP should be strengthened through improved communication strategies, including collaboration with ART clinics, health facilities, and community networks. Information on registration processes, locations, and distribution schedules should be widely disseminated using media platforms and community outreach initiatives.

### 5.3. Improve Confidential and Stigma‐free Registration

The registration process should prioritize confidentiality to reduce stigma and encourage more PLHIV to come forward.

### 5.4. Extend Support to Hard‐To‐Reach and Vulnerable Groups

House‐to‐house registration should be expanded, particularly for individuals who face physical, social, or economic barriers such as older adults, people with disabilities, and bedridden clients.

## 6. Study Contributions

This study contributes valuable empirical evidence to the limited body of literature on the intersection of nutrition support and HIV treatment outcomes in Namibia. By examining the experiences and perspectives of the stakeholders in the Khomas FSP, the study deepens the understanding of how structured nutritional assistance enhances treatment adherence, strengthens well‐being, and mitigates socioeconomic challenges faced by vulnerable clients on ART. It also highlights context‐specific barriers such as food insecurity, stigma, and mobility constraints that influence access to support services. Furthermore, the study provides practical insights for policymakers and program implementers on designing more responsive, client‐centered nutrition interventions. Overall, these findings expand the existing knowledge on integrated HIV care models and offer evidence‐based guidance for strengthening community‐level support systems in resource‐limited settings.

**Nomenclature**
AIDS: Acquired Immune Deficiency Syndrome
ART: Antiretroviral therapy
HIV: Human immunodeficiency virus
KRC: Khomas Regional Council
MOHSS: Ministry of Health and Social Services
PLHIV: People living with HIV
TB: Tuberculosis

## Author Contributions

Linda Ndeshipandula Lukolo and Martin Shapi designed and developed the study and collected and analyzed the data. Linda Ndeshipandula Lukolo and Edith Hamukwaya prepared the original draft of this paper. Linda Ndeshipandula Lukolo, Martin Shapi, and Edith Hamukwaya revised and edited the manuscript, and they did the proofreading and finalized the paper.

## Funding

No funding was received for this manuscript.

## Disclosure

All the authors approved the manuscript.

## Conflicts of Interest

The authors declare no conflicts of interest.

## Supporting Information

Additional supporting information can be found online in the Supporting Information section.

## Supporting information


**Supporting Information** Supporting 1. 1. Figure 1: A map of Namibia showing Khomas Region constituencies ((a) location of Namibia in Africa, (b) location of Windhoek in Namibia, and (c) Khomas Region and constituencies). Supporting 2. 2. Table of corrections.

## Data Availability

The datasets generated during the current study are available from the corresponding author upon reasonable request.
