# Supplementary material for: Perspectives on the Impacts of a Food Support Program on the Well‐Being of People Living With HIV (PLHIV) and on Antiretroviral Therapy (ART) in Khomas Region, Namibia
Source: AIDS Res Treat. 2026 Jul 1;2026:6201140. doi: 10.1155/arat/6201140 (PMC13323837; doi:10.1155/arat/6201140)
Supplement: Supplementary file 1 — Supporting Information Supporting 1. 1. Figure 1: A map of Namibia showing Khomas Region constituencies ((a) location of Namibia in Africa, (b) location of Windhoek in Namibia, and (c) Khomas Region and constituencies). Supporting 2. 2. Table of corrections. [file ARAT-2026-6201140-s001.docx]

**Table of corrections**

Perspectives on the Impact of a Food Support Program on the Well-being of People Living with HIV on Antiretroviral Therapy in Khomas Region, Namibia.

| Commends | Corrections |
| --- | --- |
| HIV positive | Changed to PLWHIV, page 3 |
| Indicate figure 1 | Indicated page 5 |
| Source of irregular income | For example selling street food (vetkoek or Kapana) at local market or mobile street vendors, casual construction work, their daily earnings vary day by day. On busy day they may earn better, however on slow days they may earn little or nothing, page5 |
| Replace participant with either a nurse etc | Replaced with stakeholders and the name of the constituency since they were all stakeholders from different constituencies |
| Remove initials from citations | Removed |
| Combine conclusion with recommendations | Combined |
| PLWHIV | Corrected to PLHIV, page 21 |
| Bolded references | Unbolded, page 24 |
| Unclear figure | Replaced with the clear one |
| Minor corrections | Attended to |
|  |  |
